# Characterization of antibiotic resistance genes in soils from agroecosystems of the Brazilian Amazon

**DOI:** 10.3389/fmicb.2025.1508157

**Published:** 2025-05-27

**Authors:** Taynara Cristina Santos Tavares, Lívia Freitas da Silva Pinto, Oscar Victor Cardenas-Alegria, Carlos William Dias Dantas, Sandro Patroca da Silva, Ana Cecília Ribeiro Cruz, Aníbal Coutinho do Rêgo, Hervé Louis Ghislain Rogez, Rommel Thiago Juca Ramos, Cristian Faturi, Adriana Ribeiro Carneiro Nunes

**Affiliations:** ^1^Center for the Valorization of Bioactive Compounds From the Amazon, Federal University of Pará, Belém, Brazil; ^2^Simulation and Computational Biology Laboratory (SIMBIC), High Performance Computing Center (CCAD), Federal University of Pará, Belém, PA, Brazil; ^3^Department of Arbovirology and Hemorrhagic Fevers, Evandro Chagas Institute—IEC, Ananindeua, Brazil; ^4^Department of Animal Science, Federal University of Ceará, Fortaleza, CE, Brazil; ^5^Institute of Animal Health and Production, Federal Rural University of Amazonia, Belém, PA, Brazil

**Keywords:** antibiotic resistance genes, bacterial diversity, mobile genetic elements, pasture soils, native forest

## Abstract

The conversion of forests to pastureland in the Amazon has increased over the years, resulting in significant impacts on ecosystem diversity, particularly on the soil microbiota. These changes affect the physical and biological properties of the soil, influencing the resistome and contributing to the selection and spread of antibiotic resistance genes (ARGs) in the soil environment. This study aimed to analyze the soil resistome under different managements in an Amazonian agrosystem. Soil samples were collected from the organic layer in forest and pasture areas within the municipality of São Miguel do Guamá, which included pastures managed with fertilization and those without the use of fertilizers. The samples underwent processing to extract genetic material and were sequenced using the Illumina platform. The sequences obtained were analyzed using bioinformatics tools to identify bacterial taxonomy and diversity. In addition, genetic annotation was performed using specialized databases to characterize functional genes, mobile elements, and resistance genes. The results showed changes in bacterial composition in pasture soils, where species such as *Staphylococcus aureus, Staphylococcus cohnii*, and *Bacillus coagulans* were more prevalent. In forest soils, differences in the composition of functional genes were detected, while soils without fertilizers exhibited a higher abundance of transposable elements. In addition, antibiotic resistance genes, such as macrolides, tetracyclines, aminoglycosides, among others, were more abundant in pasture soils.

## 1 Introduction

In recent decades, agricultural production has driven the expansion of pastures resulting in an annual increase of 8 million hectares of such areas globally (Gonçalves et al., 2020; Chen et al., 2020). By 2020, the approximate extent of forest land cover was 4.06 billion hectares (FAO, 2020), while 2 billion hectares were used for pastures (López-Bedoya et al., 2022). Currently, forests have been reduced by up to 50%, resulting in a loss of biodiversity and environmental impacts (Lopes et al., 2020). In Brazil, the Amazon rainforest covers 49% of the territory; however, this proportion has been decreasing over the years due to the expansion of agribusiness activities (Cardenas Alegria et al., 2022). Of the 154 million hectares of pastures in the national territory, ~65% show signs of intermediate to severe degradation (Paes da Costa et al., 2024).

The transformation of forests into pasture and crop areas in the Amazon causes changes in the soil ecosystem, characterized by the removal of vegetation and the use of chemical substances such as fertilizers, pesticides, and antibiotics (Silva et al., 2022). The increase in contaminant residues in ecosystems is conceptualized as an imbalance conducive to developing opportunistic pathogenic microorganisms, promoting the selection of antimicrobial resistance genes (Paes da Costa et al., 2022; Venturini et al., 2025).

The mechanisms of horizontal gene transfer between bacteria play a crucial role in the evolutionary dynamics of the resistome (Tokuda and Shintani, 2024) and can be influenced by environmental factors and physicochemical characteristics of ecosystems. Therefore, these aspects should be considered when analyzing the diversity of bacterial resistance (Lemos et al., 2021).

Studies show that the most commonly found antibiotic resistance genes (ARGs) are associated with tetracyclines, sulfonamides, and fluoroquinolones (Zhang et al., 2021). These drugs can persist in the soil for extended periods (Wang et al., 2020) and induce the presence of ARGs, which can be acquired by various pathogens in the soil (Zhang et al., 2024).

In this sense, next-generation sequencing (NGS) technology and metagenomics have been widely used to investigate the microbiota of specific environments, such as soil (Zhang et al., 2021). These advanced approaches facilitate the construction of metagenomic libraries and the thorough analysis of genetic material from environmental samples, providing insights into microbial diversity, population structure, genetic relationships, and environmental interactions (Behera et al., 2020a). With the help of algorithms, it is possible to improve the taxonomic profile and genetic prediction of microbial species, essential information for assessing the public and animal health risks associated with ARGs (Collis et al., 2024; Paes da Costa et al., 2024; Rout et al., 2022; De Abreu et al., 2021; Behera et al., 2020b).

On the topic of metagenomics, research has correlated knowledge between the impacts of climate change and soil microorganisms, as well as meta-analyses on the effects of crops on this ecological niche (Daugaliyeva, 2024; Pellegrinetti et al., 2024; Venturini et al., 2025). Researchers have been particularly interested in analyzing changes in soil microbial diversity along gradients of management intensity in pastures and forests. The metagenomic approach has enormous potential in studying these two agrosystems (Vieira et al., 2021).

This study aims to characterize the composition of bacterial communities and the profiles of antibiotic resistance genes (ARGs) in soils from agroecosystems under different management practices. Characterizing these genes allows for understanding the distribution of bacteria that may impact human health and identifying potential patterns of ARGs associated with soil management types. The study develops detailed information on bacterial diversity and antibiotic resistance genes selected by different management practices applied to agroecosystems, which could present different compositions due to ecosystem management.

## 2 Materials and methods

### 2.1 Site description and soil sampling

Soil samples were collected in the municipality of São Miguel do Guamá, located in the northeast of the State of Pará, Brazil. According to the Köppen classification, the region is characterized by a hot and humid climate (HF), with average annual temperatures of around 26.7°C (Alvares et al., 2013). The predominant soils in the region, accounting for ~82.1%, belong to the Yellow Latosol group, characterized by medium texture, low natural fertility, high acidity, and intense leaching (Leite et al., 2023).

Three different soils were considered for the study of the agrosystem: native forest (NF; −1.492534, −47.629718); pasture with treatment 1 (PT1), corresponding to soil managed with the use of fertilizers (−1.507812, −47.646530); and pasture with treatment 2 (PT2), referring to soil without the use of fertilizers (−1.494170, −47.643097). The characteristics of each site are described in Table 1, and the location of the collection site is illustrated in Supplementary Figure S1.

**Table 1:** Statistical data on biodiversity and description of the different locations.

**Location**	**Description**	**Alpha-diversity index of bacteria**		
		**Richness**	**Shannon**	**Simpson**
Native forest (NF)	At the equatorial tropical forest site, commercial timber was only removed around 20 years ago.	5,077.333 ± 1.527	6.663 ± 0.373	0.983 ± 0.138
Pasture treatment 1 (PT1)	The area was cleared using burning and stump removal, and the development of different pasture species was left in place, having been used for over 10 years. Pasture with larger coverage of grasses from the *Megathyrsus* genus is designated for breeding cows and is managed under rotational grazing with annual fertilization (limestone and chemical fertilizers).	5,081 ± 1	6.782 ± 0.189	0.989 ± 0.809
Pasture treatment 2 (PT2)	The area was cleared using burning and stump removal, and the development of different pasture species was left in place, having been used for about 8 years. The pasture, with larger coverage of grasses from the *Urochloa* genus, is intended for calf rearing and is managed under rotational grazing, without fertilization or soil correction.	5,082.333 ± 2.816	5.318 ± 1.258^*^	0.984 ± 0.809

^*^p < 0.05.

The soil samples were collected following a zigzag pattern on the site's surface to obtain a representative sample of the study area. Three sampling points were selected for each experimental condition, located 50 m apart. Five holes were drilled using a Dutch auger at each point, generating initial subsamples. The subsamples from the five boreholes at each point were combined to form a composite sample representative of that point. Three subsamples were taken from each composite sample, resulting in nine subsamples. These subsamples were used for biological analysis and stored in 50-ml Falcon tubes, which were kept in liquid nitrogen. In addition, the quartering technique was applied to ensure that the soil's physical, chemical, and molecular analyses were representative of each sampling point.

### 2.2 Physical and chemical soil analysis

For the chemical analysis of the soil from the three sites, 50 g of samples from each repetition were mixed. The samples from each site were dehydrated in an oven at 40°C with air circulation and passed through a sieve with a 2.0-mm diameter mesh. The following parameters were determined: organic matter (OM), pH measured in aqueous suspension, calcium (Ca^2+^), and magnesium (Mg^2+^). As for the physical analysis, sieving and sedimentation techniques were used to calculate the fractions of sand, silt, and clay present in the soil, where the soil was sieved through a series of sieves with decreasing openings (2, 0.5, 0.2, 0.125, and 0.053 mm), and the fractions retained on each sieve were dried and weighed to determine the particle size distribution; The data obtained was used to calculate the percentage of each fraction, allowing the size of each particle of the material analyzed to be characterized (Teixeira et al., 2017).

### 2.3 DNA extraction and sequencing analysis

DNA extraction was carried out using the commercial DNeasy PowerSoil kit (Qiagen, USA), following the protocol guidelines provided by the manufacturer. After extraction, the samples were stored appropriately at −20°C. The quality of the extracted genetic material was assessed using a NanoDrop ^®^ spectrophotometer (NanoDrop–Thermo Fisher Scientific), and only samples with concentrations higher than 50 ng/μl and purity levels in the range of 1.8–2.0 were accepted. To assess the integrity of the extracted DNA, electrophoresis was carried out on a 1% agarose gel with Tris-acetate-EDTA (TAE) buffer and 0.5 μg/ml ethidium bromide.

The samples were processed according to the manufacturer's protocol of Nextera XT DNA Library (Illumina) to build the DNA library. Sequencing was performed using the NextSeq 550 System High-Output Kit for a read size of 2 × 150. The platform used was the Illumina NextSeq 550 High Output, and the entire process was carried out according to the manufacturer's protocol.

The quality of the reads obtained by triplicate sequencing was first analyzed using the FASTQC version 0.11.9 (Saheb Kashaf et al., 2021). They were then trimmed and filtered with a minimum quality standard of Phred 20 by the Trimmomatic (Sewe et al., 2022).

### 2.4 Taxonomic analysis

The pre-treated readings were subjected to taxonomic analysis using the Kraken2 software (Wood et al., 2019), with the PFP (Plants, Fungi and Protozoa) database, which contains comprehensive information on a wide range of microorganisms, including those present in environmental samples. The comparative matrix of the taxonomic classifications of the organisms was built using the Pavian platform (Breitwieser and Salzberg, 2020).

The MicrobiomeAnalyst 2.0 platform (Lu et al., 2023) was used to assess the diversity and microbial composition of the samples. The relative abundance of the data was normalized using the trimmed mean of the M values (TMM) technique. In the analysis of the alpha-diversity indices, the Simpson and Shannon Richness indices were considered, and for beta-diversity, the Analysis of Similarity of Distances (ANOSIM) and Non-metric Multi Dimensional Scaling (NMDS) statistical approaches were applied. The bacterial taxonomic composition was also analyzed at the taxonomic levels of phylum, family, and species using the same platform as above.

### 2.5 Determination of resistance genes and mobile genetic elements

Reads were assembled using MEGAHIT 2.4.3 (Li et al., 2015), with specific settings for soil metagenomic data (parameter: meta-large); the resulting contigs were analyzed with the MetaQuast 3.2 (Mikheenko et al., 2016) to assess quality after assembly. The Prokka 1.2 (Seemann, 2014) was used to identify the coding regions.

The following parameters were applied to obtain information on functional genes: an e-value of 10^−5^, a minimum identity of 60%, and a minimum sequence length of 15 nucleotides (nt). The analysis was performed using the SEED Subsystems database through the Metagenomic Rapid Annotations using Subsystem Technology (MG-RAST) version 4.0.3 (Meyer et al., 2019), focusing on identifying mobile genetic elements (MGEs).

Antimicrobial resistance genes (ARGs) were identified by processing the clean reads using CARD-Resistance Gene Identifier (CARD-RGI) 4.0.2 (Alcock et al., 2023). Subsequently, the files were selected to perform abundance transformations according to the method described by Inda-Díaz et al. (2023). Similarly, genes with 70% coverage were selected, followed by the selection of the features “mechanism” and “drug class.”

### 2.6 Statistical analysis

The results were subjected to the Shapiro–Wilk normality test, with a significance level of 5% (*p* < 0.05). The data obtained by chemical and physical analysis, including particle size distribution and alpha-diversity between communities, were subjected to analysis of variance (ANOVA) or Kruskal–Wallis test using the PAST 5.2 (Hammer et al., 2001). Python (Bouzenia et al., 2024) was then used to generate graphs, using libraries such as Matplotlib, Pandas, and Seaborn.

## 3 Results and discussion

### 3.1 Physicochemical characteristics and microbial diversity in the soil

The physicochemical analysis of the different study sites revealed the following information about the components. Starting with pH, we observed a pattern of acidity in all the sites between high and medium, with values in NF of 4.53, in PT1 of 4.91, and in PT2 with values of 5.22. According to the concentration of organic matter (OM), there was variation between the sites, with NF showing the highest concentration (16.86 g kg^−1^), followed by PT2 (15.14 g kg^−1^) and the lowest proportion at site PT1 (14.00 g kg^−1^). Calcium ions (Ca^2+^) were low in all the sites, but with the proportions in the NF sites (1.39 cmolc dm^−3^), followed by PT1 (1.13 cmolc dm^−3^), and the lowest in PT2 (0.26 cmolc dm^−3^). In contrast, magnesium ion (Mg^2+)^ concentrations were also low at all sites, showing a different distribution with PT1 (0.32 cmolc dm^−3^), followed by NF (0.18 cmolc dm^−3^) and PT2 (0.07 cmolc dm^−3^). When forest soils are modified for pasture or crop use, their physicochemical parameters change due to the transformation in ecosystem composition (Amorim et al., 2020; De Lima et al., 2022; Pessôa et al., 2023). Consequently, changes at the micro-ecosystem level are important to understand how these influence physicochemical characteristics (Yang et al., 2019).

In relation to pH, acidity values were found to be between strong and moderate, with higher values at site PT2, with NF being considered a strongly acidic soil, as has also been reported in the soil of the Brazilian Atlantic Forest (Teixeira et al., 2017; Vazquez et al., 2020).

In terms of OM content, considerably higher values were found in the NF and PT2 sites. However, another study found that pasture soils had higher OM values than forest soils (Walkup et al., 2020). Even so, managed pasture soils could have better soil properties (De Lima et al., 2022), which could be considered a recurring practice to prevent soil degradation. Paes da Costa et al. (2024) pointed out that the highest Ca^+2^ levels were observed in pasture areas with high fertilization, possibly due to the addition of compounds such as limestone. However, higher levels were not identified in pasture soils compared to forest soils in our study. Similarly, Mg^+2^ levels tend to be high in sites with grass cover (Momesso et al., 2022). Our study found higher concentrations of this cation in soils with more *Megathyrsus* grass cover and where fertilizer was applied. This result suggests that the management type can directly influence the soil's magnesium levels.

When analyzing the granulometry data, we identified a larger presence of fine sand in the pasture soils, while the NF soil showed similar proportions of fine and coarse sand. In addition, all the samples had identical amounts of clay (Supplementary Figure S2).

Previous studies indicate that the granulometric composition of soils generally reveals a higher proportion of sand in relation to clay, which corroborates our results (Giongo et al., 2022). However, Barrezueta Unda et al. (2019) found different proportions, with larger amounts of silt in forest soils and high concentrations of clay in pasture areas.

Particle size analysis is essential for understanding soil characteristics, especially in sensitive ecosystems such as sandy pasture soils with lower water retention. However, clay in these soils contributes to nutrient retention, favoring the development of microorganisms (Eftene et al., 2022).

With regard to the alpha-diversity indices, we observed that the average richness showed minimal differences between the pasture and forest sites. The Shannon index values were higher in the PT1 and NF soils, in contrast to the PT2 soil, with significant differences (ANOVA; *p* < 0.00). Similarly, Simpson's index recorded values with subtle differences between them (Table 1).

This disparity in alpha-diversity indices in pastures supplemented with fertilization was documented by Paes da Costa et al. (2024), where adding fertilizers could influence the recovery of microbial diversity (Giongo et al., 2022). Thus, bacterial diversity and species richness are significantly affected by the lack of appropriate management in pasture soils (Melo et al., 2021). The diversity indices of PT1 were almost equivalent to NF soils, indicating a possible influence on soil quality and the recovery of vegetation cover, reflecting on bacterial communities (Damian et al., 2021).

The beta-diversity analysis revealed an apparent grouping pattern, forming possible clusters between the replicates of the different samples (NMDS, stress = 0.019), as illustrated in Supplementary Figure S3. However, the ANOSIM analysis indicated low group differentiation (*R* = 0.358; *p* = 0.019).

Our findings indicate significant ecological differences between sites, suggesting that bacterial composition is highly sensitive to environmental factors. This reinforces the idea that bacterial communities vary according to local conditions (Rout et al., 2022). Furthermore, our results are consistent with those of Das et al. (2024), who highlighted the influence of physicochemical parameters in structuring these communities.

### 3.2 Composition of bacterial communities in the soil

The yield of readings in different locations was highest in the pasture soil samples with treatment 1, with an average of 6,340.3563 readings. This was followed by pasture soil with treatment 2 with 430,814.15 readings, and forest soil samples with 424,894.57 readings.

The taxonomic classifications showed the following yield: between 85.7 and 87.3% of the readings obtained were unclassified, while between 12 and 20.4% of the readings obtained were used for classification. Of these, an average of 17% were identified as bacteria in the PT1 soil, 13.73% in the PT2 soil, and 12.90% in the forest soil.

**Figure 1:**
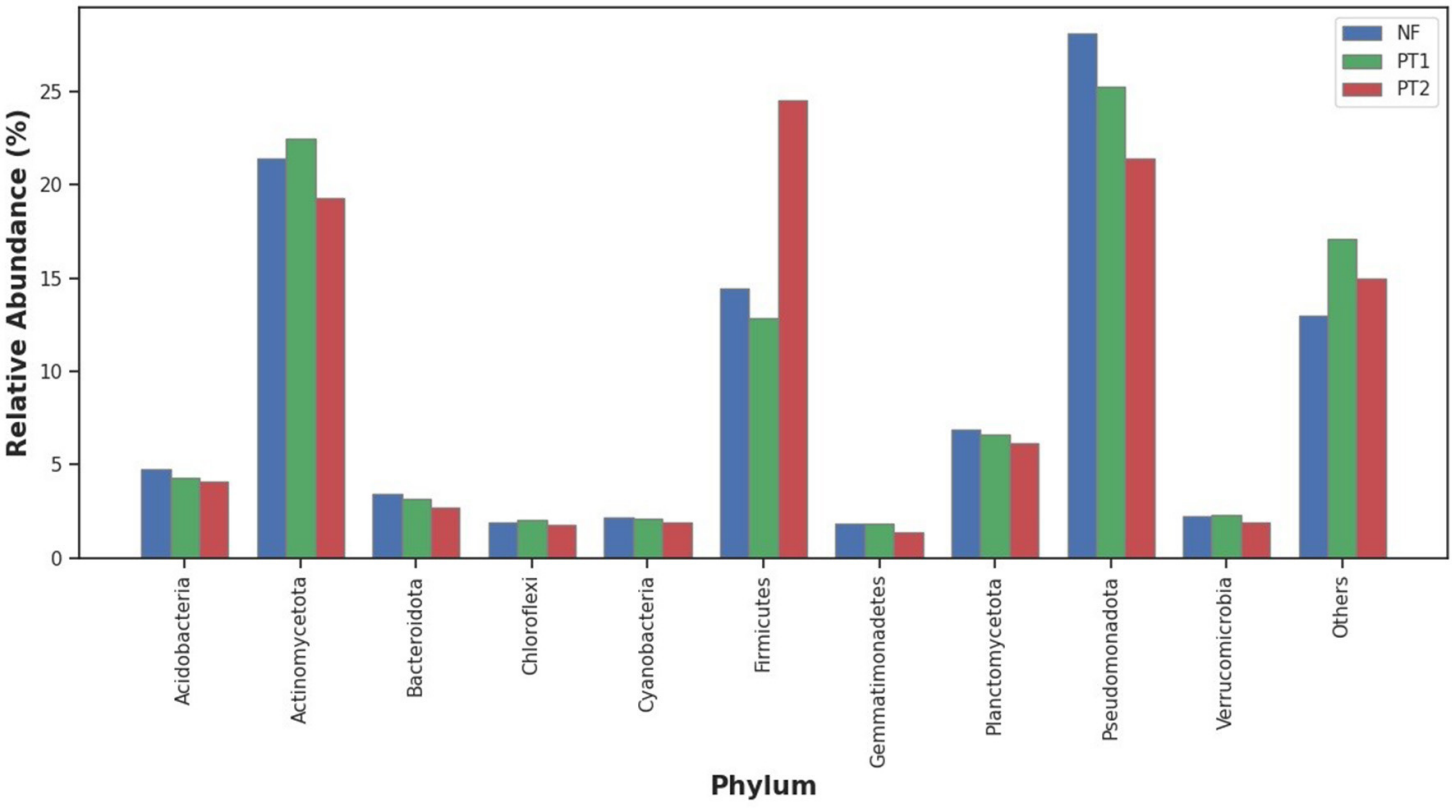
Distribution of abundance of the taxonomic classification at the phylum level of the bacteria domain in the different locations under study.

Analysis of the taxonomic classification at the phylum level showed that the NF and PT1 soils had a predominance of the *Pseudomonadota* phylum, followed by *Actinomycetota* and *Firmicutes*. Still, the proportion in the PT2 soil was higher for the latter (Figure 1).

In other studies, the compositions at the phylum level in forest soil stand out *Acidobacteria* and *Verrucomicrobia*, but in soils of managed and cultivated pastures, they presented *Pseudomonadota, Bacteroidetes, Firmicutes*, and *Gemmatimonadetes* (Walkup et al., 2020; Tomazelli et al., 2023).

Forest soils, characterized by a high availability of organic matter, often favor a larger abundance of the *Actinomycetota* phylum (Idbella and Bonanomi, 2023), which was also observed in this study. In contrast, in pasture soils, appropriate management and correction can positively influence the abundance of microorganisms. In contrast, pasture soils without fertilization and/or correction tend to show a reduction in microbial abundance, due to degradation and lower nutrient availability (Golovchenko et al., 2023; Zhou and Wang, 2023).

However, soils with larger coverage by grasses of the *Urochloa* genus, commonly used as forage, may influence the composition of the bacterial community. Previous studies suggest that this type of vegetation cover may be associated with a higher abundance of *Firmicutes* (Araujo et al., 2023). In the present study, a higher abundance of this phylum was observed in the PT2 soil, which may be related to this factor, although this association was not directly tested. Similarly, pasture soils, without fertilization and correction, tend to accumulate more organic matter, favoring the abundance of this phylum, which has better adaptation in these environments and can withstand stressful conditions (Pedrinho et al., 2019).

**Figure 2:**
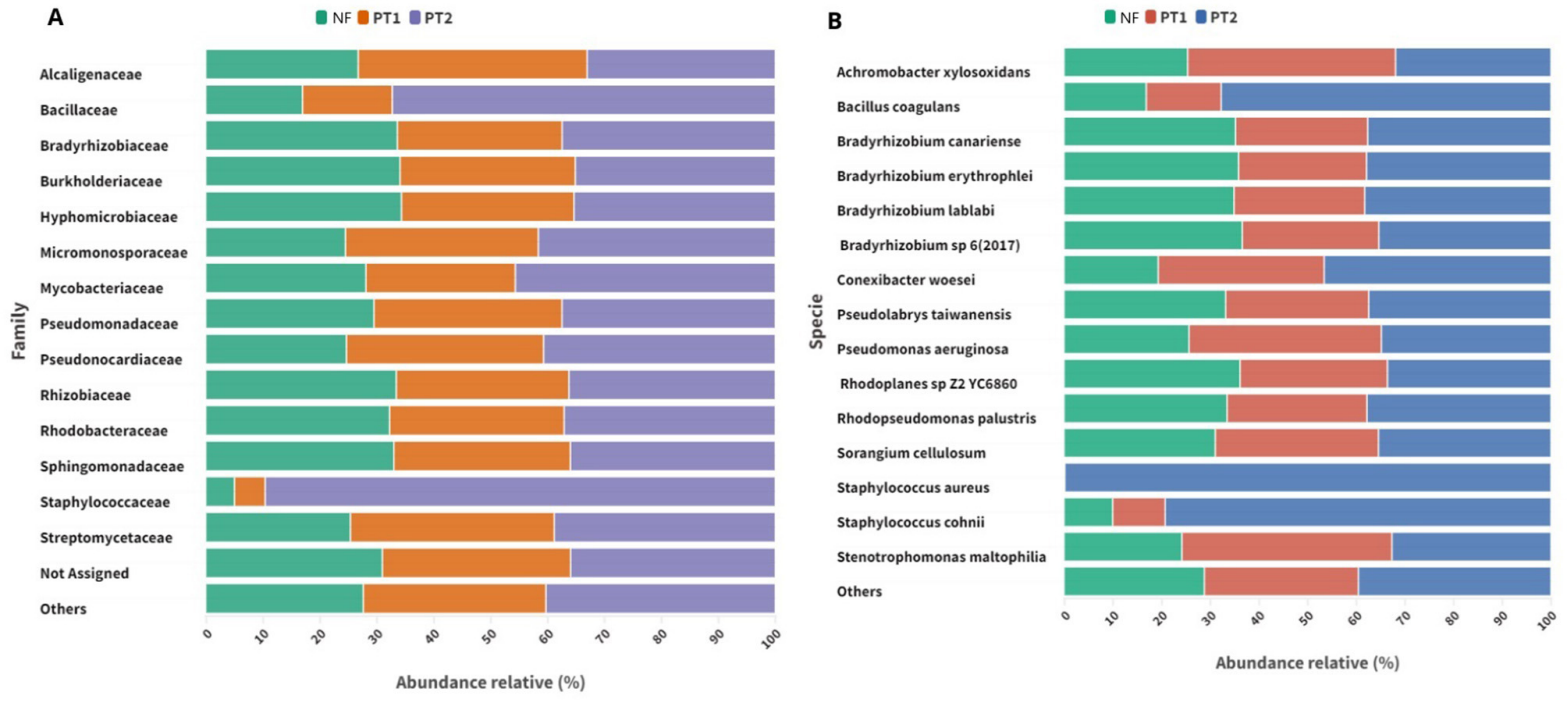
Bacterial composition at the taxonomic level of **(A)** family and **(B)** species in different locations.

The taxonomic analysis at the family level showed a larger predominance of *Staphylococcaceae* and *Bacillaceae* in the PT2 soil compared to the other sites (Figure 2A). At the species level, the presence of *Staphylococcus aureus* stood out, followed by *S. cohnii* and *Bacillus coagulans* in soil PT2. In addition, *Pseudomonas aeruginosa* was detected at all the sites studied (Figure 2B).

The dominant vegetation in pasture soils can play a fundamental role in determining microbial communities. Some grass genera release specific root exudates that can favor the proliferation of certain bacterial groups, such as the Staphylococcaceae family (Brisson et al., 2019). These can provide substrates that serve as a carbon and energy source for certain bacteria, creating an environment conducive to their growth (Tomaszewska et al., 2023).

Another essential factor to consider is using fertilizers, a common practice in agricultural management, which can stress microbial populations over time, inhibiting or eliminating some bacterial groups (Acharya et al., 2021). In contrast, the absence of these inputs may require larger adaptation of bacterial communities to maintain ecosystem stability (Van Der Bom et al., 2018).

Recent studies indicate that inadequate soil management methods can favor the spread of *S. aureus* strains in agricultural environments (Babin et al., 2019; Kozajda et al., 2019). In contrast, the abundance of *S. cohnii* may be associated with specific environmental conditions that favor its development, this being a potentially pathogenic opportunistic bacterium (Park and Ronholm, 2021; Dincă et al., 2022). In this study, the presence of these species was evidenced in pasture soils, especially at site PT2.

Pasture degradation is a process that results in a continuous decrease in productivity due to inadequate management practices, leading to a loss of soil fertility and a reduction in vegetation cover, which can result in soils with lower nutrient availability and larger exposure to environmental stress factors (Dias-Filho, 2017). In this context, an abundance of bacteria such as *B. coagulans*, known for their ability to form resistant spores, can be favored, as their spores allow them to survive in adverse conditions and compete effectively for limited resources (Guimarães et al., 2022).

In general, microbial composition can serve as an indicator of soil quality, as it quickly reflects the effects of changes in ecosystems, portraying changes in metabolic functions (Fierer, 2017). The loss of microorganism diversity can have profound impacts on nutrient cycling in the soil, altering the geometabolic reactions that occur for the proper functioning of the ecosystem (Paes da Costa et al., 2024).

### 3.3 Functional analysis

**Figure 3:**
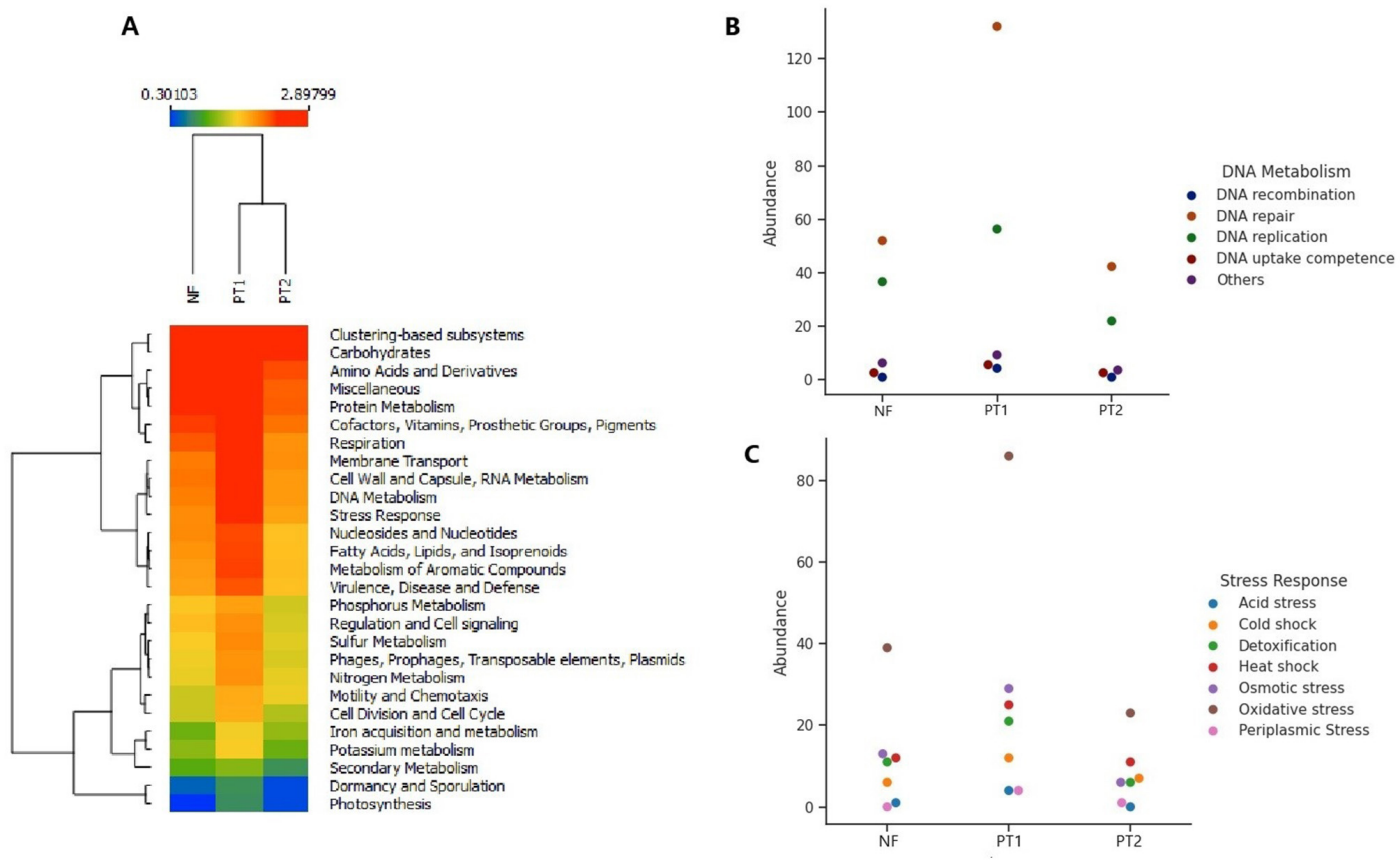
Composition of functional genes classified in the SEED Subsystems database, **(A)** classification at level 1, genes in the classification at level **(B)**. DNA metabolism and **(C)** stress response.

The evaluation of functional genes across the different sites revealed variations in their composition and abundance, with PT1 presenting the highest values compared to the other locations (Figure 3A), showing significant differences among them (ANOVA; *p* = 0.0004). Tukey's *post-hoc* test indicated that the PT1 group differs significantly from FN (*p* = 0.00976) and PT2 (*p* = 0.00046), while FN and PT2 did not show a statistically significant difference between them (*p* = 0.6038).

It was also possible to observe groupings according to the relative abundance of the genes: low, moderate, and high. The high abundance group identified genes related to protein and carbohydrate metabolism, amino acids and derivatives, membrane transport, and various functions, as well as virulence and stress. The moderately abundant group includes genes associated with cell regulation and signaling, sulfur, nitrogen and phosphorus metabolism, mobile elements, and others. Finally, the high abundance group contains genes linked to photosynthesis, dormancy, and sporulation.

The findings about functional genes revealed that the soil microbial communities showed distinct functional profiles, especially between grassland soils with different management. They reflect the adaptations of microbial communities to the specific conditions of each habitat, influenced by factors such as plant diversity and management practices (Li et al., 2020).

The highest abundance of genes related to carbohydrate metabolism was in the PT1 site, suggesting that these microbial communities are most needed to degrade the different substrates, such as cellulose and hemicellulose from plant remains, that are in this site (Castañeda and Barbosa, 2017).

In turn, different biological processes were highlighted, such as the analysis of DNA metabolism, where the results indicated variations in the abundance of various categories of genes between the three sites, with a larger abundance of DNA repair genes at the PT1 site (Figure 3B), which could indicate a prominent activity of stress factors at these sites, with a high abundance of genes associated with oxidative stress (Figure 3C).

The use of fertilizers in PT1 soil could influence the development of oxidative stress and DNA damage. Thus, amended soils supplemented with fertilizers and other products can affect plant development and soil microdiversity (Rodrigues et al., 2023). Similarly, heavy metals in fertilizers can damage the genetic material of microbial communities (Afshana and Reshi, 2021; Chen et al., 2020). Prolonged fertilization significantly increases the rate of soil respiration. It is associated with the level of oxidative stress (Seixas et al., 2022), and the existence of genetic material repair genes helps microorganisms maintain DNA integrity and adapt to these adverse conditions (Beltran-Garcia et al., 2021). However, it was not possible to carry out the analysis on soil samples that had different fertilizers, pesticides, and other biocides. Including these factors would allow for a more detailed understanding of the stress and repair mechanisms highlighted, offering valuable information on how these agents influence microbial communities and soil resilience.

### 3.4 Mobile genetic elements (MGE)

**Figure 4:**
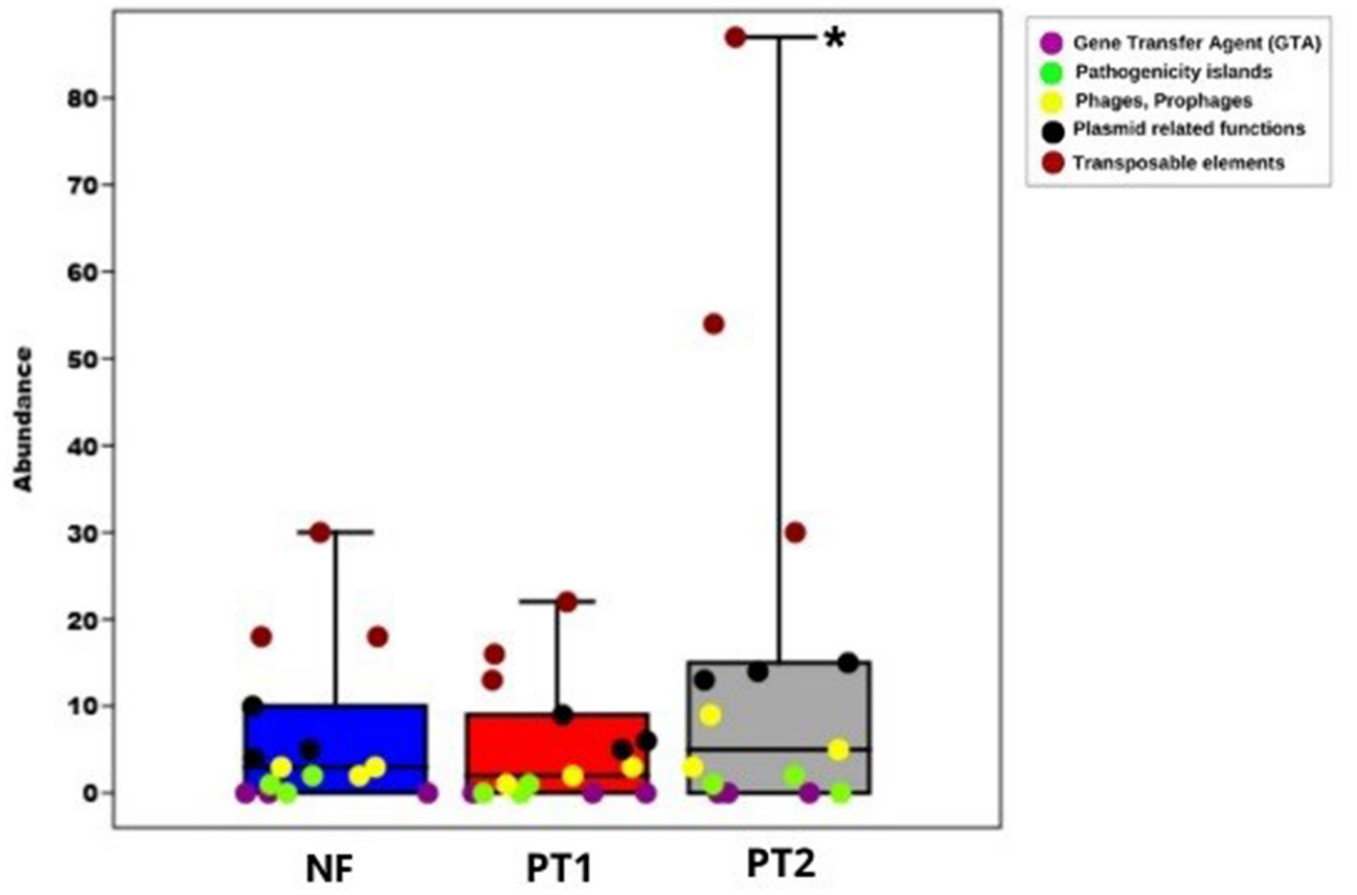
Box plot of mobile genetic elements and their components distributed in different locations (*p < 0.05).

Despite the lack of statistically significant differences between the groups (*p* > 0.05), a higher abundance and variability of mobile genetic elements, particularly transposable elements, were observed in the PT2 group (Figure 4), suggesting possible activation or mobilization of these elements in this environment. This trend may reflect larger environmental stress or selective pressure in PT2, potentially promoting genomic plasticity.

Recent studies have highlighted the role of transposable elements in genomic plasticity and the adaptation of various organisms. For example, a genomic analysis of *Ralstonia solanacearum* revealed over 10,000 insertion sequences, demonstrating the contribution of these elements to the pathogen's diversity and evolution (Gonçalves et al., 2020). Although this study is not directly related to the PT2 group, it reinforces the relevance of transposable elements in bacterial genome dynamics. Furthermore, other studies have shown that distinct environments can influence the distribution and abundance of mobile genetic elements, with larger microbial diversity associated with such elements, possibly favoring horizontal gene transfer (Greenblum, 2024; Vale et al., 2022).

Sites with larger environmental stress, due to biotic and abiotic factors, can stimulate the transfer of genetic material through transposable elements (TE), which provide mutations and promote recombination, facilitating adaptation in these ecosystems (Liang et al., 2021; Weisberg and Chang, 2023). This was identified at site PT2, which shows slight soil degradation, indicating that environmental stress can play an essential role in the genetic dynamics of microbial communities.

Horizontal gene transfer is one of the primary mechanisms highlighted for the adaptation of microorganisms, and conjugation is one of the processes that facilitates the transfer of different genes (Roquis et al., 2021). Our samples also showed the presence of this type of plasmid, which suggests that conjugation may contribute to the genetic dynamics observed in microbial communities.

### 3.5 Analysis of antibiotic resistance genes (ARGs)

**Figure 5:**
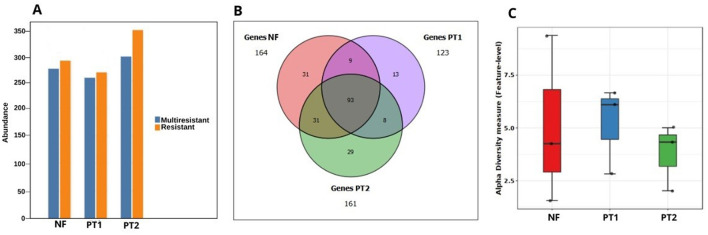
**(A)** Distribution of resistance/multiresistance in the resistome in soils with and without pasture. **(B)** Euler diagram of the presence of resistance genes. **(C)** Fisher's alpha-diversity analysis of antimicrobial resistance genes in different locations.

The analysis of antibiotic resistance genes generally identified the presence of resistance to different antibiotics, mainly at the PT2 site (Figure 5A). In the case of the analysis of ARGs, a total of 214 genes were identified in the three sites, 93 of which are shared between all of them. In addition, there were exclusive genes for each site: NF has 31 genes, followed by PT2 with 29, and to a lesser extent, at the PT1 site, with 13 genes (Figure 5B). With regard to the ARG genetic diversity index, the highest values were found in NF and PT1 (Figure 5C).

The presence of shared genes between the three sites suggests that there are native bacterial communities with a significant group of genes that perform relevant functions in each environment. Due to changes in ecosystems, there has been a diversification and specialization of microorganisms to adapt to these environmental conditions (Miles et al., 2019; Lemos et al., 2021). As a consequence, there may also be site-specific genes, reflecting the local adaptation of microbial communities.

Genetic diversity is essential for responding to different stress factors (Salgotra and Chauhan, 2023), such as the natural antibiotics present, especially in forest soils, which contain several molecules analogous to antibiotics (Mohan et al., 2023). For this reason, the genetic diversity index is expected to be high in NF soil, reflecting the adaptation of bacterial communities to these natural compounds.

Recent studies have shown that inadequate soil management, such as excessive use of chemical fertilizers, lack of crop rotation, and absence of soil treatment, can favor the selection of resistant bacteria and the spread of ARGs (Huang et al., 2021). In addition, poor management practices increase leaching and surface runoff, leading to the contamination of water bodies and other environmental compartments (Neher et al., 2020; Pan et al., 2023). This spread represents a significant threat to public health by facilitating human and animal exposure to resistant bacteria, including through contaminated food (Almeida et al., 2023), among the different opportunistic pathogens, the genus *Staphylococcus* was observed, which are present in urban and rural ecosystems and pathogens from rural areas were considered high risk (Li et al., 2023), as was identified in pasture sites. Inadequate management practices could also promote the persistence of resistance genes in the soil, which may help explain their higher abundance in areas like PT2, where there is no fertilization or soil correction.

On the contrary, sustainable management practices, such as proper composting of organic waste, implementation of vegetative buffer zones, rational use of antimicrobials in livestock, and continuous soil quality monitoring, have been identified as practical strategies to mitigate these risks (Keenum et al., 2021; Rehman et al., 2022). These measures can reduce not only the microbial and genetic load in soils but also minimize impacts on aquatic ecosystems and local biodiversity.

**Figure 6:**
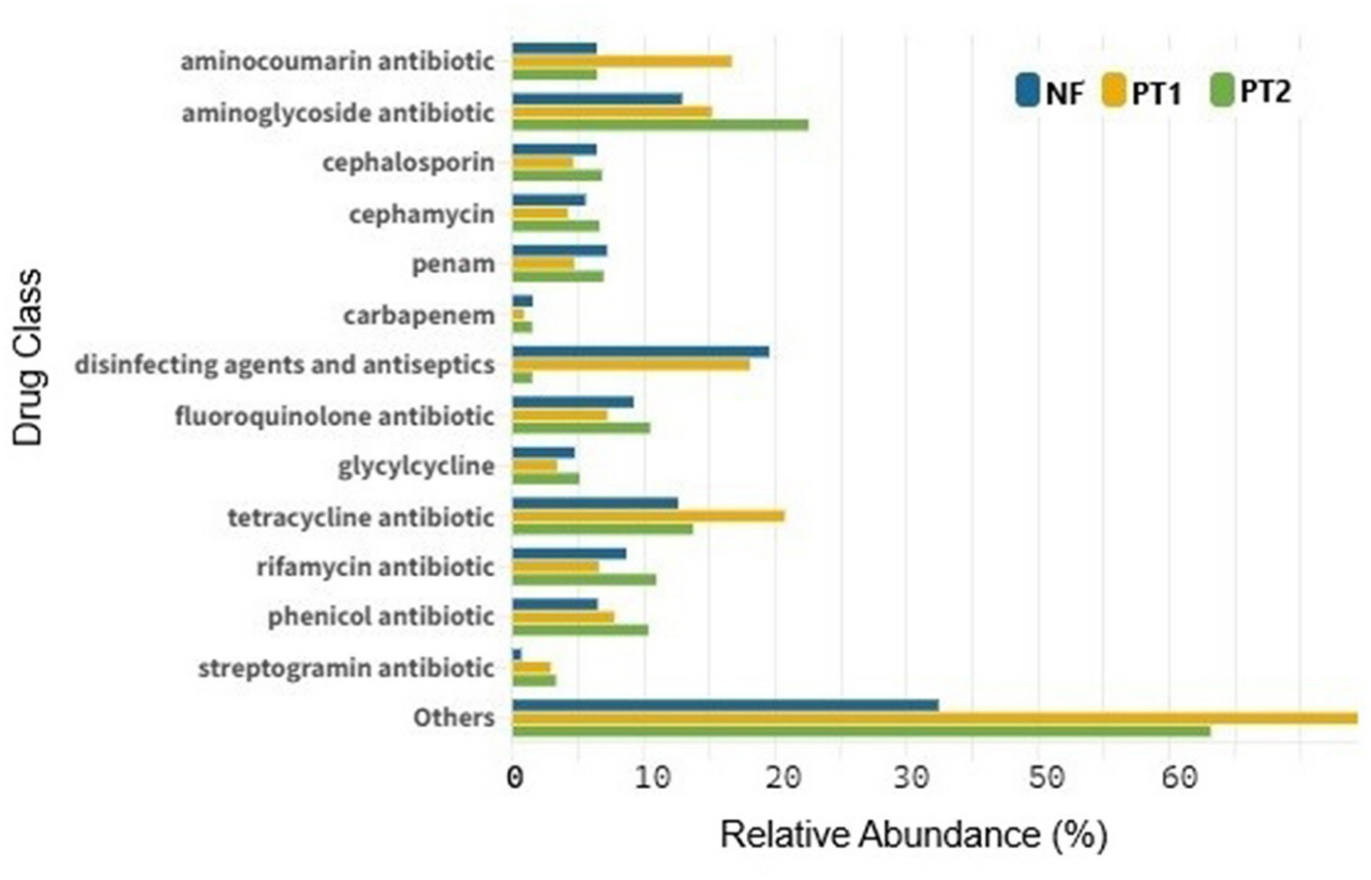
Abundance of antibiotic-resistant drug classes from different locations.

With the ARGs identified, it was possible to observe the presence of resistance to various classes of antibiotics, with a higher abundance of macrolides, especially at the PT2 site. When excluding the extreme abundance of this class, resistance to other antibiotics was observed, such as tetracycline, aminocoumarin, as well as disinfectants and antiseptics, which were predominant at the PT1 site. The latter class was also prominent in the NF soil, while resistance to aminoglycosides prevailed at the PT2 site (Figure 6). These findings align with other studies highlighting resistance in pastures (Cardenas Alegria et al., 2022; Lawther et al., 2022).

Animals do not fully absorb antibiotics used by the veterinary industry, leaving active residues in urine and feces (Jia et al., 2023). This is the case with the abundance of aminocoumarins, aminoglycosides and tetracyclines present in soil (Pitta et al., 2020; Rothrock et al., 2021), which could explain the presence of resistance genes in these places. However, Li et al. (2020), found a higher abundance of ARGs in soils treated with manure, which could be considered a potential reservoir of ARGs (Wang et al., 2020). In this context, animal manure possibly contains antibiotics and microorganisms with ARGs, contributing to the spread of antibiotic resistance. Although the PT2 site does not receive fertilizers, the larger presence of manure than PT1 may explain the abundance of these drugs and genes in these soils.

The use of disinfectants and antiseptics in agricultural environments, although effective against microorganisms that cause infections, can have important implications for bacterial resistance. According to the article by Nagati et al. (2021), the inappropriate or prolonged use of agents such as povidone-iodine and hydrogen peroxide can favor resistance in bacteria. In addition, the uncontrolled and irresponsible use of these products can favor the selection of resistance genes in bacteria present in the environment, negatively impacting the local microbiota and contributing to the persistence of these agents in soil and water (James et al., 2023). In soils such as those in NF, compounds analogous to biocides can favor the natural selection of resistance genes, creating an environment conducive to the proliferation of resistant microorganisms (Ferreira et al., 2024). In contrast, in soils such as PT1, the abundance of disinfectant and antiseptic agents could be associated with the excessive sanitary management implemented in these areas to prevent diseases in cattle (Silva et al., 2020).

With regard to antibiotic resistance mechanisms, the results indicate similar profiles at all sites. Target site alteration was the most prevalent mechanism at all sites, followed by efflux pumps and target site protection (Supplementary Figure S4). Our results corroborate previous studies that have identified antibiotic deactivation mechanisms such as efflux pumps and cell protection strategies in forest and grassland soils (Zhang et al., 2019; Qian et al., 2021). Regarding the abundance of resistance genes, the *EF-Tu* gene was predominant in *Escherichia coli* at the three study sites, which belongs to the *elfamycin-resistant EF-Tu* gene family. In addition, at the PT2 site, the *qacG* gene, which is part of the Small Multidrug Resistance (SMR) family, stood out (Supplementary Figure S5a). However, subtracting the genes mentioned above as extreme data, it was possible to identify the abundance of 12 different genes at the sites, of which the *adef* and *MuxA* genes stood out, both belonging to the resistance nodulation cell division (RND) family, see Supplementary Figure S5b.

The abundance of *E. coli EF-Tu* indicates the bacterium's adaptive capacity in varied environments; its high abundance can maintain the efficiency of protein synthesis even under stressful conditions, such as nutrient availability and temperature (Harvey et al., 2019).

The *qacG* gene stands out for its abundance, especially at the PT2 site. This gene is associated with resistance to quaternary ammonium compounds (QACs), which are widely used in the veterinary, medical, and industrial sectors, including cationic surfactants, antiseptics, herbicides, and lipophilic dyes (Quan et al., 2023). The high concentration of the *qacG* gene in the soil could be attributed to the intensive use of disinfectants containing QACs. These compounds play a fundamental role in livestock farming, widely applied to sanitize facilities, equipment, and animals (Li et al., 2024).

Other genes with larger abundance were *adeF* and *MuxA*, both of which were involved in resistance to multiple antibiotics through an efflux system, and these antibiotics are used in different clinical treatments for various infections by human and animal pathogens (Luo et al., 2021). The spread of these genes in farming environments can be attributed to the frequent use of antibiotics and disinfectants in livestock farming, which exerts selective pressure favoring bacteria carrying efficient efflux systems, leading to the selection of resistant strains (Checcucci et al., 2020).

Analysis of the core genes of the ARGs identified at the different sites revealed a larger presence of the RND efflux pump family, with a larger presence at the grassland sites. In addition, it was observed that the composition of the core genes identified at the PT1 site was different. In contrast, the compositions of these genes at the NF and PT2 sites showed similarities (Supplementary Figures S6a–c).

Increased exposure to antimicrobials due to human activity favors the selection of resistance mechanisms such as efflux pumps. A high number of antibiotic resistance genes may indicate larger selective pressure in the environment (Liu et al., 2022). In this way, we can suggest that the difference in genetic composition between the NF and PT1 sites, despite having different ARG profiles, maintains similar genetic diversity values. This may indicate the influence of pasture soil management on the distribution and maintenance of these resistance genes.

However, identifying different resistance genes in this type of sample can be subject to biases, since various factors can influence the results. These include the quality of the genetic material, the sequencing platform used, and possible errors in the development of the methodology (Długosz and Deorowicz, 2024), which can impact the detection and quantification of resistance genes.

Similarly, the database used can also influence the detection of these genes. In the case of CARD-RGI, this database stands out from others because of its specific characteristics (Papp and Solymosi, 2022) and because it provides more detailed information for studying the resistome, which is why it was used in this study. However, the presence of genes that have not yet been fully characterized, as occurs in other databases (Gschwind et al., 2023), this could affect the underestimation of the genes found. Similarly, an alternative to improve gene identification could be to use a combination of different databases. However, the challenge lies in standardizing the databases' information in the case of MEGARes (Bonin et al., 2023). Third-generation sequencing platforms, which generate larger products, would help reduce the errors indicated above, and they may be alternative platforms to be used in these types of samples (Chen et al., 2024).

### 3.6 ARG coexistence analysis

**Figure 7:**
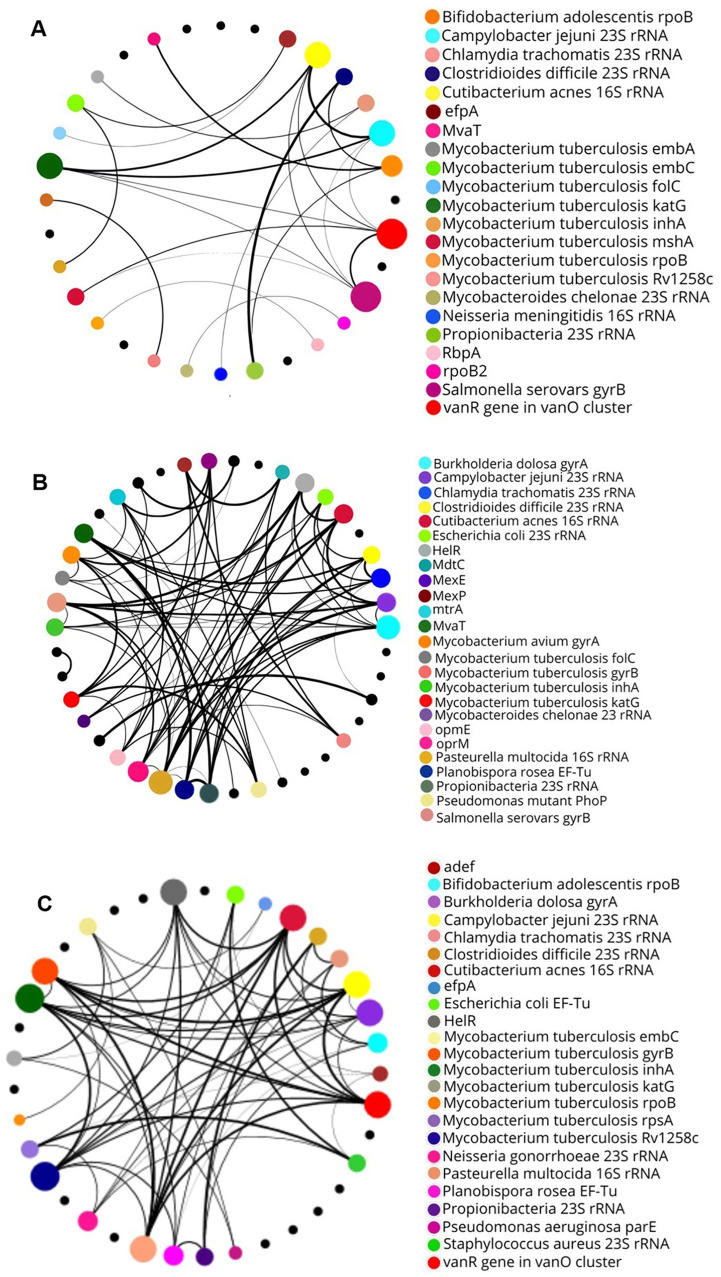
Correlations between bacterial genes by network analysis. **(A)** In soil NF, each gene presents up to five interactions. **(B)** In soil PT1, each node presents up to six interactions. **(C)** In soil PT2, up to nine interactions are observed between genes.

Analysis of the coexistence of the most abundant ARGs at the different sites revealed unique characteristics for each environment. Thus, the NF site showed a low number of gene interactions (Figure 7A). In contrast, the greatest number of interactions between the genes were between the pasture sites, especially at the PT2 site, with up to nine interactions at just one node, which corresponds to the *Cutibacterium acnes* gene 16S rRNA mutation conferring resistance to tetracycline (Figures 7B, C). Studies such as that by Cheng et al. (2020) indicate that agricultural and pasture environments favor denser interactions between ARGs, especially in places with larger human intervention. Given this, it is plausible that mutations conferring resistance to tetracycline could arise in *C. acnes* or other bacteria present in pasture soils, especially in areas subject to selective pressures such as antibiotics. The results reinforce the influence of the type of pasture management on the coexistence of these genes. In the case of the NF soil, the low abundance of similar genes and the limited connections may be related to the transitory presence of molecules analogous to antibiotic classes. This factor may restrict the proliferation of these genes in the short term. Thus, environmental conditions play a key role in the maintenance and distribution of these genes (Cassan et al., 2021).

The pasture sites showed a larger connection between the antibiotic resistance genes, reflecting a high coexistence between these genes. However, further research is needed to confirm this hypothesis and better understand the mechanisms involved. This could improve understanding of the simultaneous occurrence of these genes in these environments, as demonstrated in studies analyzing the presence of multiple genes associated with different phenotypes and environmental conditions (Nagpal et al., 2020).

In recent years, significant advances have been made in the development of molecular tools for detecting and quantifying ARGs in various ecosystems. Among these technologies, microarray and multiplex digital PCR stand out, which enable more detailed and comprehensive analyses, allowing the simultaneous identification of different genes under different environmental conditions (Ouyang et al., 2024; de la Cruz Barron et al., 2023).

## 4 Conclusion

In the analysis carried out in this study, it was possible to find differences between the different sites, with higher proportions of fine sand in the pasture soils. In the case of the alpha-diversity indices, lower values were found in the pasture site without the use of fertilizers, where the presence of *S. aureus* bacteria was highlighted, followed by *S. cohnii* and *B. coagulans*.

Similarly, in these pasture soils, the presence of mobile elements and resistance genes against the macrolide and aminoglycoside classes of antibiotics stood out. In contrast, forest and pasture soils with fertilizer showed resistance to the disinfectant and antiseptic agent classes, followed by the tetracycline and aminocoumarin antibiotics. In addition, the analysis of gene coexistence showed larger interactions in the pasture sites, especially in sites without the use of fertilizers.

Therefore, practices adopted in pasture soils, especially when there is a lack of proper management with fertilizers and soil correction, can significantly influence bacterial and genetic diversity, including antibiotic resistance genes (ARGs). These changes not only affect soil quality but also pose potential risks to animal and human health. These changes can impact soil quality and animal and human health. Proper soil management practices, such as controlled fertilizer use, pH correction with lime, and rotational grazing, are essential to maintaining soil health. Our findings reinforce the importance of public policies aimed at sustainable pasture and agroecosystem management. Adopting guidelines that consider the dynamics of the soil resistome is essential to curb the spread of antibacterial resistance and reduce its impacts on a regional and global scale.

## Data Availability

The datasets presented in this study can be found in online repositories. The names of the repository/repositories and the accession number(s) can be found at: https://dataview.ncbi.nlm.nih.gov/object/PRJNA1214040?reviewer=e5n9ae7iievchuj72r2nm47ddf.
