# Supplementary material for: Characterization of antibiotic resistance genes in soils from agroecosystems of the Brazilian Amazon
Source: Front Microbiol. 2025 May 27;16:1508157. doi: 10.3389/fmicb.2025.1508157 (PMC12149118; doi:10.3389/fmicb.2025.1508157)

Supplementary Material

1. Supplementary Figures

Supplementary Figure 1. Map of the location of the farm where soil samples were collected, corresponding to the municipality of São Domingos do Capim, in the state of Pará, Brazil.

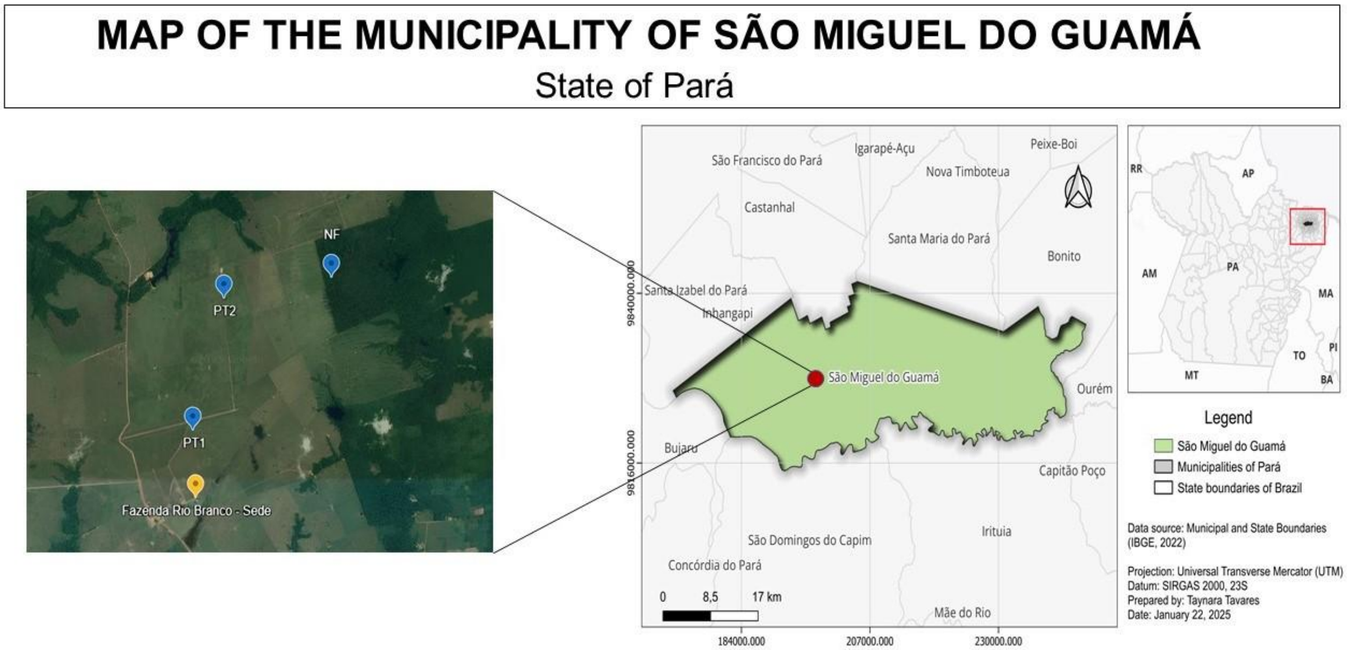

Supplementary Figure 2. Grain size distribution in the soil at different sites.

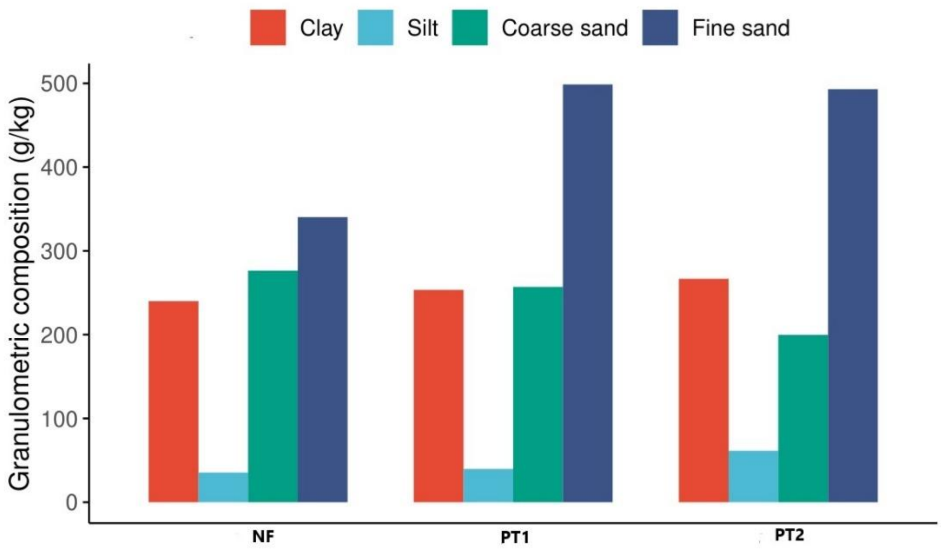

Supplementary Figure 3. NMDS analysis of bacterial samples from different sites

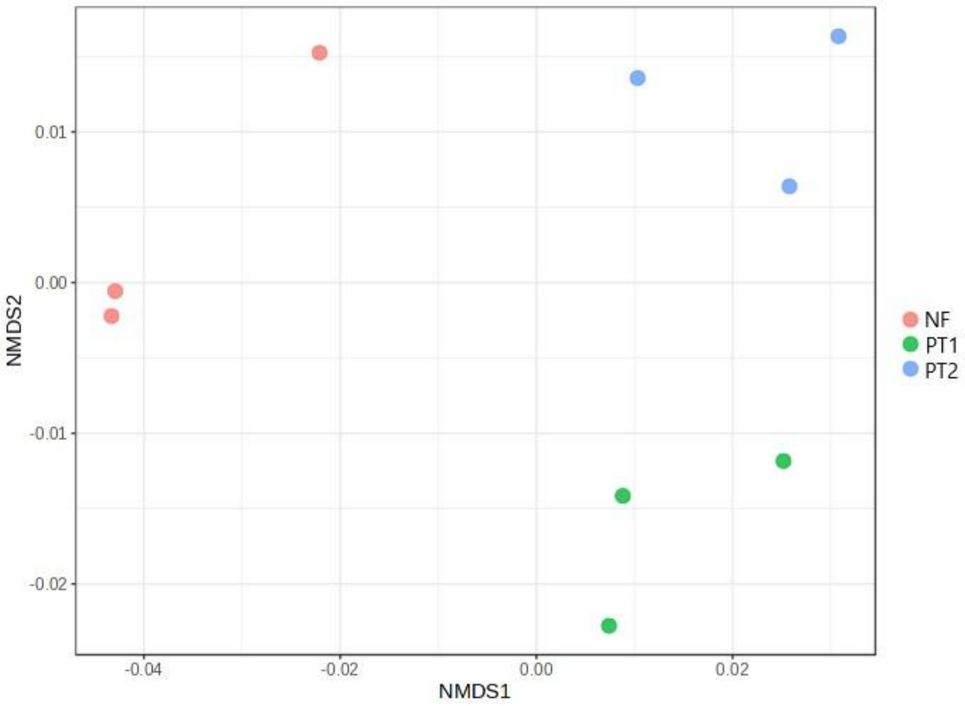

Supplementary Figure 4. Arrangement of antimicrobial resistance mechanism classification across different study sites.

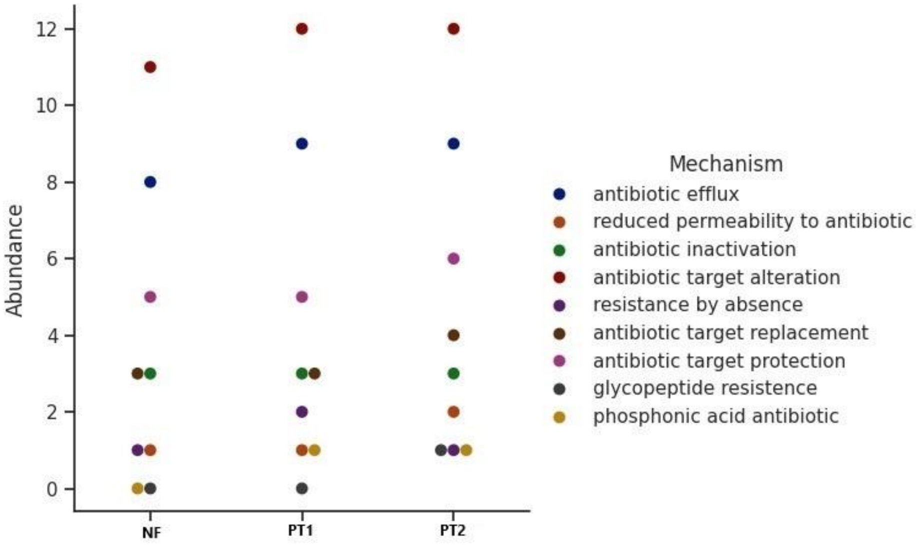

Supplementary Figure 5. Relative abundance of resistance genes at different sites. A. Predominance of the *Escherichia coli EF-Tu* gene at all three study sites. B. The *adeF* and *MuxA* genes stand out after removal of outlier data.

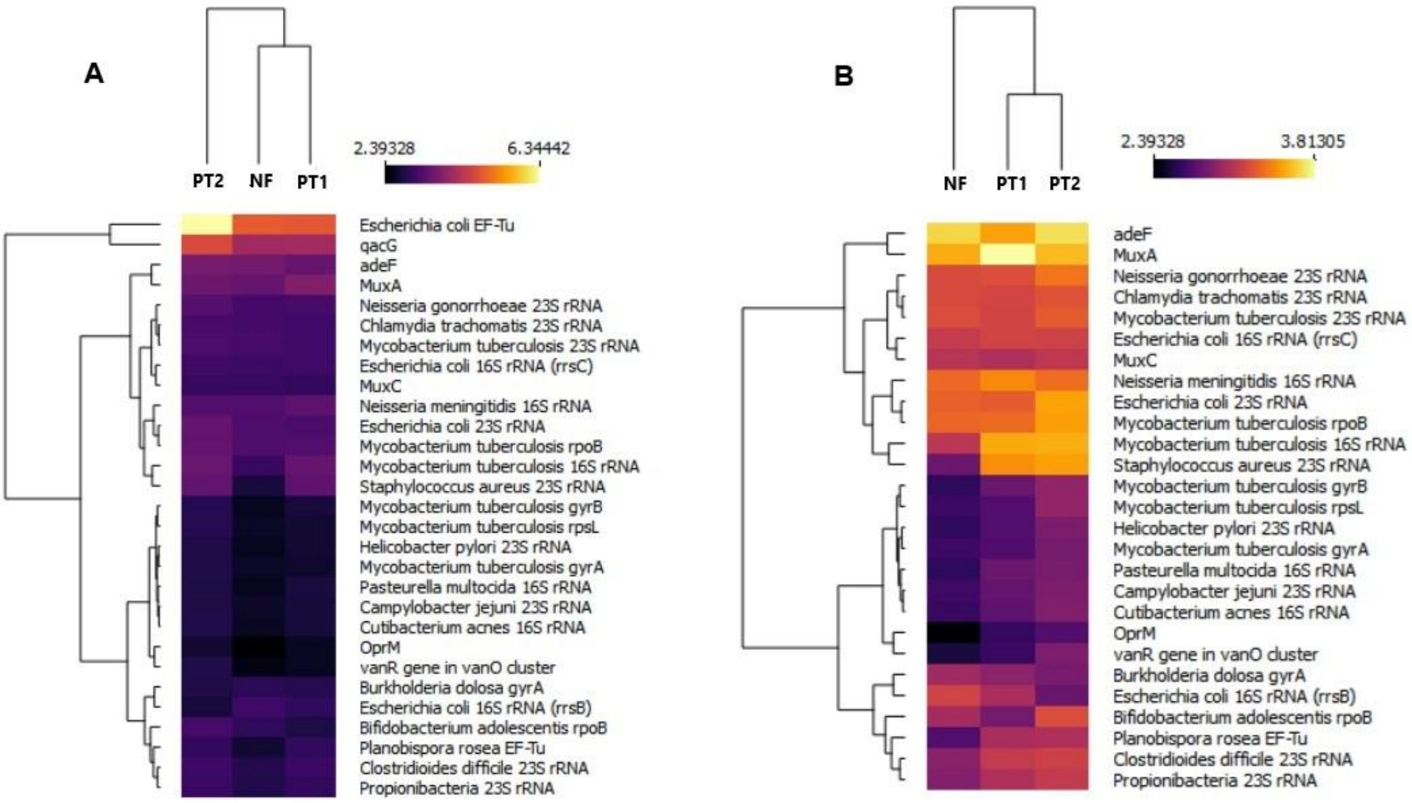

Supplementary Figure 6. Core distribution of the relative abundance of resistance genes across different sites.

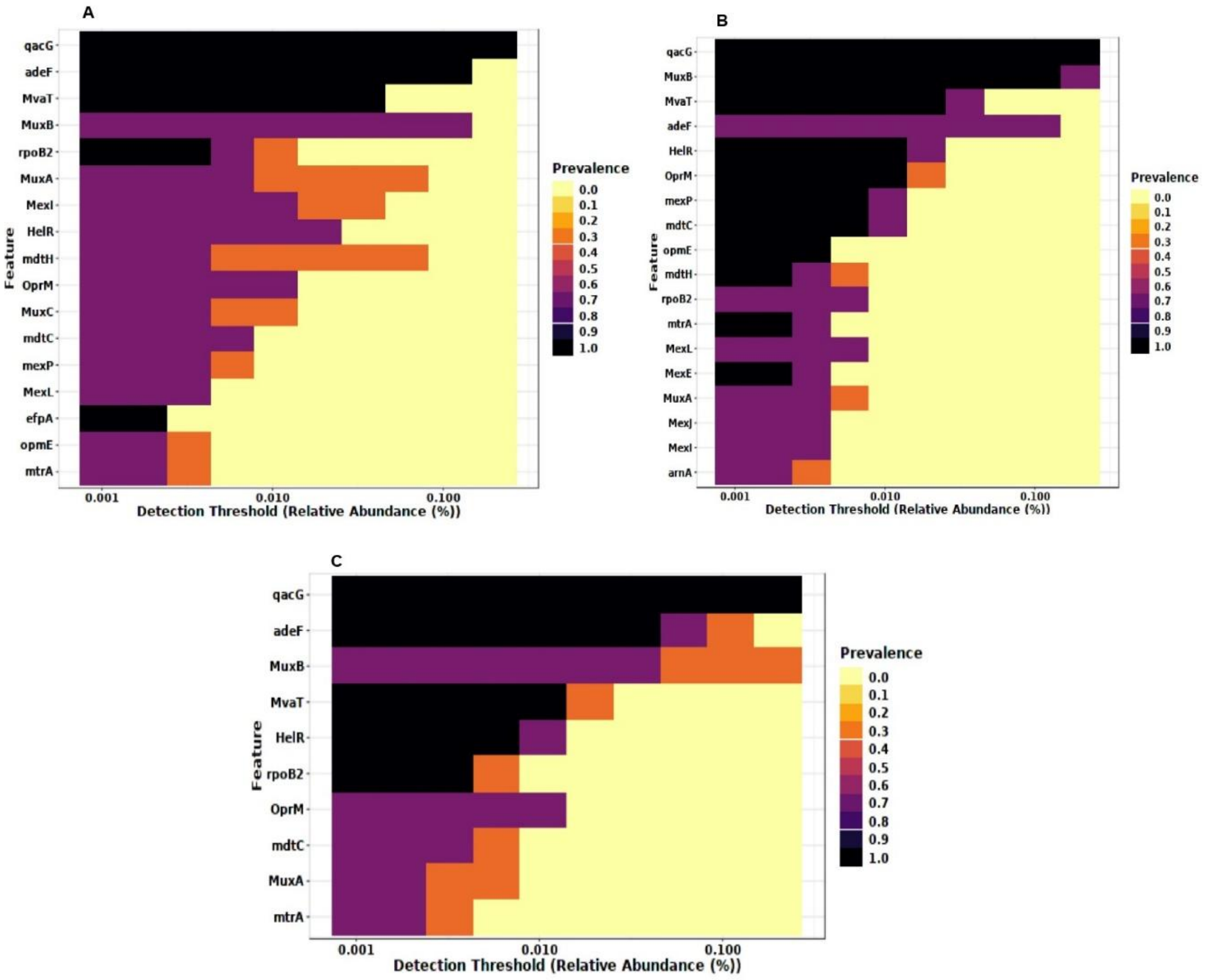

Supplement: Supplementary file 1 [file Data_Sheet_1.pdf]
